# The importance of intravenous immunoglobulin treatment in critically ill patients with necrotizing soft tissue infection: a retrospective cohort study

**DOI:** 10.1186/s12879-022-07135-6

**Published:** 2022-02-21

**Authors:** Daniel A. Hofmaenner, Pedro David Wendel Garcia, Manuel R. Blum, Sascha David, Reto A. Schuepbach, Philipp K. Buehler, Pascal M. Frey, Annelies S. Zinkernagel, Silvio D. Brugger

**Affiliations:** 1 Institute of Intensive Care Medicine, University Hospital Zurich, University of Zurich, Raemistrasse 100, 8091 Zurich, Switzerland; 2 Department of General Internal Medicine, Bern University Hospital (Inselspital), University of Bern, Bern, Switzerland; 3 Institute for Primary Health Care (BIHAM), University of Bern, Bern, Switzerland; 4 Department of Infectious Diseases and Hospital Epidemiology, University Hospital Zurich, University of Zurich, Zurich, Switzerland

**Keywords:** Necrotizing soft tissue infections, Intravenous immunoglobulins, Decision making, Combination therapy

## Abstract

**Background:**

Necrotizing soft-tissue infections are infections with high mortality. The use of immunoglobulins within a combination therapy including broad-spectrum antibiotics has been debated. We assessed potential benefits of immunoglobulins and hypothesized that they were associated with a treatment benefit in a high-resource setting.

**Methods:**

Patients with necrotizing soft-tissue infection hospitalized in the tertiary intensive care unit of the University Hospital of Zurich, Switzerland, between 2008 and 2020 were included retrospectively. The association between immunoglobulin administration and in-hospital survival, intensive care unit length of stay, the incidences of acute renal failure, acute respiratory distress syndrome and septic shock were analyzed.

**Results:**

After adjustment for confounders, no difference for in-hospital survival (hazard ratio 2.20, 95% confidence interval [CI] 0.24–20.20, p = 0.5), intensive care unit length of stay (subhazard ratio [SHR] 0.90, CI 0.41–1.98, p = 0.8) and the development of acute respiratory distress syndrome (SHR 1.2, CI 0.36–4.03, p = 0.77) was observed in patients with or without immunoglobulin treatment. The Simplified Acute Physiology Score II, the risk of developing acute renal failure (SHR 2.86, CI 1.33–6.15, p = 0.01) and septic shock (SHR 1.86, CI 1.02–3.40, p = 0.04) was higher in patients treated with immunoglobulins, possibly reflecting a higher disease severity beyond measured confounders.

**Conclusions:**

No clear evidence for a benefit of immunoglobulins in our cohort with consistent antibiotic use was found. Patients receiving immunoglobulins appeared more severely ill. Complementary to high treatment standards and appropriate antibiotics including beta lactams and protein synthesis inhibitors, immunoglobulins should be administered on a case-to-case basis, at least while more evidence from larger randomized controlled trials is missing.

## Background

Necrotizing soft-tissue infections (NSTI) are severe infections with high morbidity and mortality [1]. Factors leading to increased mortality include age, comorbidities (diabetes, immunosuppression and cardiovascular diseases), delay in antibiotic treatment or surgical intervention [2–5]. Early diagnosis and immediate treatment of NSTI including resuscitation, fasciectomy and antibiotics are of paramount importance [6]. In order to reduce the bacterial burden as well as virulence factor activity, protein synthesis inhibitors are recommended together with beta lactam antibiotics.

The use of intravenous immunoglobulins (IVIG) to treat NSTI has been debated in recent years. In theory, IVIG neutralize microbial toxins and antigens, have immunomodulatory functions and facilitate bacterial opsonisation [7].

Due to the fulminant nature and rarity of the disease, prospective clinical trials have been difficult to conduct. Clinical studies investigating benefits of IVIG in NSTI have yielded controversial results because of their non-interventional or non-randomized design, or relatively small sample size [8–11]. Murine necrotizing fasciitis models, however, showed that IVIG attenuate virulence factor activity of group A streptococci and reduce disease severity [12]. In light of remaining uncertainties and limited available evidence in relation to the use of IVIG in NSTI, their standard use is currently not recommended by the Infectious Diseases Society of America [13].

To investigate patient-centred clinical outcomes from more than a decade of experience in treating patients with NSTI at our tertiary intensive care unit (ICU), we aimed to retrospectively assess potential benefits of our IVIG treatments, reflecting real-life clinical management. We hypothesized that administration of IVIG was associated with an additional treatment benefit in our high-resource setting with a high standard of care.

## Methods

### Study design and cohort sample

In this retrospective cohort study, consecutive patients with NSTI aged ≥ 18 years hospitalized in the tertiary ICU of the University Hospital of Zurich, Zurich, Switzerland, between 2008 and 2020 were included. Patients were included in the analysis if NSTI was confirmed intraoperatively according to the surgical reports and if NSTI and synonymous terms “necrotizing fasciitis”, “Fournier’s gangrene” and “necrotizing cellulitis” were the main diagnosis in the billing of the ICD-10 codes irrespective of the microbiological results.

Exclusion criteria were either incomplete or implausible electronic medical records, or when the responsible clinicians in charge of the patient disagreed with the diagnosis of NSTI according to the medical records.

The relevant ethics committee approved the study (Kantonale Ethikkommission Zurich BASEC-ID 2016-00145 and 2017-02225). Permission to obtain and access relevant data was included as a part of the formal ethics approval and was approved by the committee. The study was conducted in accordance with the Helsinki Declaration.

### Baseline data collection

For enrolled patients baseline data was collected using the in-hospital medical records database (KISIM Version 5.0, Cistec AG, Zurich, Switzerland). Baseline data included demographics, the Simplified Acute Physiology Score II (SAPS II) [14], substance abuse (smoking, alcohol abuse, drug abuse), intake of steroids and other immunosuppressive drugs, and comorbidities.

Additionally, data for the probable etiology of NSTI (trauma, skin lesion, drug injection, intramuscular injection, hematogenic, animal/insect bite, others), the affected body region and body surface area, the microbiological type (polymicrobial, monomicrobial, unknown) and the surgical and antibiotic treatment were collected. Laboratory values at admission were also included.

### IVIG treatment and study outcomes

The used polyclonal IVIG (Privigen®) were composed of a pooled (> 1000 donors), non-targeted, purified formulation containing at least 98% immunoglobulin G (IgG). The standard IVIG treatment was IVIG 1 g/kg body weight at admission, followed by IVIG 0.5 g/kg body weight for the following two days. The pre-defined study outcomes were the association of IVIG treatment with five clinical outcomes. The primary outcome was in-hospital survival, further outcomes were ICU length of stay, the incidences of acute renal failure [15], acute respiratory distress syndrome ARDS [16] and septic shock [17] according to the medical reports.

### Statistical analysis

The association of IVIG treatment and mortality was examined using a Cox proportional hazards model. For the investigation of IVIG treatment and further clinical outcomes a competing risk regression model according to Fine and Gray was used to account for the competing risk of death [18] to accommodate for a potentially high mortality in this population. Selection of confounders to adjust the model was restricted to one variable per 10 patients [19]. Outcomes and variables included in the models were selected according to their presumed clinical relevance.

Analyses were performed using SPSS Version 23 (SPSS Science, Chicago, IL, USA) and Stata 16 (Stata Corporation, College Station, TX, USA).

## Results

### Study sample

**Fig. 1:**
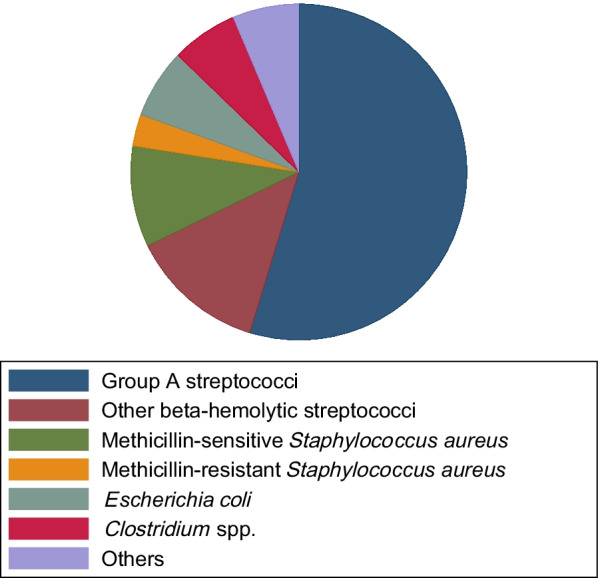
Isolated microorganisms from patients with monomicrobial etiology of NSTI (31 of 48 patients). The two detected *Clostridium* spp. were *Clostridium perfringens* and *Clostridium septicum*

We included 48 patients in this study, of whom 22 were treated with IVIG. Patients receiving IVIG tended to be younger, more often female and had a higher SAPS II score. Otherwise, patients were similar in terms of baseline characteristics and treatment modalities (Table 1). Overall, 35 (72.9%) survived their hospital stay. Median ICU length of stay was 11.5 days (interquartile range [IQR] 4 to 20 days) whereas length of hospital stay was 28.5 days (IQR 19 to 44.5 days). Acute renal failure occurred in 31 (64.6%) of patients. Incidences of ARDS and septic shock were 11 (22.9%) and 29 (60.4%), respectively. Overall, 31 out of 48 patients (64.6%) presented with a monomicrobial etiology of NSTI (Fig. 1). 17 patients (35.4%) demonstrated a monomicrobial group A streptococcal infection. The other causative microorganisms (monomicrobial) are depicted in Fig. 1. Table 2 shows the cumulative causative microorganisms stratified by IVIG treatment. The causative microorganisms are summarized in pathogen groups. In patients not treated with IVIG, a cumulative number of 44 microorganisms were identified. In patients treated with IVIG, 35 microorganisms were identified (Table 2).

**Table 1:** Baseline parameters, comorbidities, etiology and disease characteristics, microbiologic type, treatment, laboratory parameters at admission and antibiotics used

	Non-IVIG (n = 26)	IVIG (n = 22)	p-value
Patient characteristics			
Age (y)	62.5 [54–71] (55–66)	51 [43–63] (44–63)	0.027
Male sex	17 (65.4%)	10 (45.5%)	0.244
Smoking	9 (34.6%)	6 (27.3%)	0.756
Alcohol abuse	5 (19.2%)	4 (18.2%)	> 0.99
Drug abuse	3 (11.5%)	1 (4.5%)	0.614
Steroids before NSTI diagnosis	4 (15.4%)	4 (18.2%)	> 0.99
Immunosuppressed	2 (7.7%)	1 (4.5%)	> 0.99
SAPS II score	37 [21–64] (26–58)	46.5 [36–69] (39–69)	0.148
Comorbidities			
HIV	0 (0%)	1 (4.5%)	0.458
Hepatitis B/C	4 (15.4%)	1 (4.5%)	0.357
Active cancer	3 (11.5%)	0 (0%)	0.239
Diabetes	7 (26.9%)	4 (18.2%)	0.514
Arterial occlusion disease	3 (11.5%)	0 (0%)	0.239
Venous insufficiency	1 (3.8%)	2 (9.1%)	0.587
Renal impairment at admission	7 (26.9%)	13 (59.1%)	0.039
Liver impairment at admission	1 (3.8%)	5 (22.7%)	0.081
Etiology of NSTI			
Trauma/wound	10 (38.5%)	5 (22.7%)	0.351
Skin lesion	7 (26.9%)	7 (31.8%)	0.758
Drug injection (iv)	1 (3.8%)	0 (0%)	> 0.99
Intramuscular injection	1 (3.8%)	0 (0%)	> 0.99
Hematogenic	3 (11.5%)	0 (0%)	0.239
Animal/insect bite	2 (7.7%)	2 (9.1%)	> 0.99
Unknown/others	2 (7.7%)	8 (36.4%)	0.152
Characteristics of NSTI			
Onset of symptoms until surgery (d)	4 [2–10] (2–9)	2.5 [2–5] (2–4)	0.164
Affected body surface area (%)	5 [2–9] (4–9)	6.5 [5–9] (5–9)	0.228
Affected body region			
Arms	5 (19.2%)	2 (9.1%)	0.429
Legs	15 (57.7%)	12 (54.5%)	> 0.99
Trunk	4 (15.4%)	6 (27.3%)	0.478
Genitals	1 (3.8%)	2 (9.1%)	0.587
Head/neck	1 (3.8%)	0 (0%)	> 0.99
Type of NSTI			
Polymicrobial	9 (34.6%)	4 (18.2%)	0.329
Monomicrobial	14 (53.8%)	17 (77.3%)	0.132
Unknown	3 (11.5%)	1 (4.5%)	0.614
Treatment			
Negative pressure wound therapy	21 (80.8%)	19 (86.4%)	0.71
Amputation	3 (11.5%)	1 (4.5%)	0.614
Scrotectomy	1 (3.8%)	0 (0%)	> 0.99
Secondary suture	7 (26.9%)	9 (40.9%)	0.366
Local flap	1 (3.8%)	2 (9.1%)	0.587
Free flap	6 (23.1%)	7 (31.8%)	0.532
Mesh-graft/euroskin transplantation	19 (73.1%)	17 (77.3%)	> 0.99
Number of reoperations	3.5 [3–7] (3–5)	5.5 [4–8] (4–7)	0.116
Laboratory parameters (admission)			
LRINEC score	6 [4–8] (6–8)	7 [5–9] (7–10)	0.255
White blood count (G/L)	14.4 [7.5–22.7] (9.2–19.7)	16.3 [8–25.2] (11.2–23)	0.569
Hemoglobin (g/dl)	11.1 [8.1–13.1] (9.1–12.6)	11.4 [9.6–12.3] (10–11.9)	0.804
Hematocrit (%)	32.4 [24.7–38.4] (26.6–35.8)	34.5 [29.5–36.1] (31.5–35.7)	0.42
Platelet count (G/L)	216.5 [142–293] (168–276)	141 [69–217] (90–217)	0.088
Alanine aminotransferase (U/L)	39 [22–70] (24–58)	49 [26–66] (30–66)	0.622
Alkaline phosphatase (U/L)	95 [49–126] (66–115)	96.5 [62–138] (62–130)	0.833
Lactate dehydrogenase (U/L)	421.5 [279.5–647.5] (325–626)	527 [425–567] (447–554)	0.593
Blood glucose level (mmol/l)	6.9 [5.4–8.3] (5.7–8)	6 [5.1–7.6] (5.3–6.6)	0.258
Urea (mmol/l)	10 [5.6–12.6] (5.8–11.8)	10.1 [7.1–17] (7.1–17)	0.4
Creatinine (umol/l)	93 [73–173] (84–124)	144.5 [92–280] (102–227)	0.03
Sodium (mmol/L)	139 [133–142] (137–142)	135.5 [133–139] (133–138)	0.093
Potassium (mmol/L)	3.9 [3.7–4.2] (3.8–4.4)	4 [3.6–4.4] (3.7–4.4)	0.633
C-reactive protein (mg/L)	217.5 [91–324] (132–305)	289 [118–350] (196–348)	0.234
Antibiotics used according to group			
Aminopenicillins	19 (73.1%)	10 (45.5%)	
Cephalosporins	12 (46.2%)	18 (81.8%)	
Carbapenems	13 (50%)	9 (41%)	
Lincosamides	21 (80.8%)	22 (100%)	
Glycopeptides	9 (34.6%)	5 (22.7%)	
Quinolones	8 (30.8%)	2 (9.1%)	
Others	6 (23.1%)	4 (18.2%)	

Data expressed as number (%) or median, [Interquartile Range] and (95% confidence interval), calculated where appropriate. Groups compared using Mann–Whitney U Test or Fisher’s exact test, as appropriate. IVIG, intravenous immunoglobulins; SAPS II, Simplified Acute Physiology Score II; NSTI, necrotizing soft tissue infection; LRINEC, laboratory risk indicator for necrotizing soft tissue infection

**Table 2:** Cumulative causative microorganisms stratified by IVIG treatment

	Non-IVIG (n = 44)	IVIG (n = 35)
Group A streptococci	6 (13.6)	14 (40)
Other streptococci	5 (11.4)	3 (8.6)
Methicillin-sensitive *Staphylococcus aureus*	8 (18.2)	1 (2.9)
Methicillin-resistant S*taphylococcus aureus*	1 (2.3)	0
*Clostridium* spp.	2 (4.5)	0
*E. coli*	5 (11.4)	3 (8.6)
Other Enterobacterales	3 (6.8)	2 (5.7)
*Pseudomonas* spp.	4 (9.1)	0
*Enterococcus faecalis*	2 (4.5)	2 (5.7)
*Acinetobacter* spp.	3 (6.8)	0
Others	5 (11.4)	10 (28.6)

Data expressed as numbers and (percentages) per group. Number of causative pathogens may be larger than number of patients due to occasional polymicrobial infection. The two detected *Clostridium* spp. were *Clostridium perfringens* and *Clostridium septicum*. IVIG, intravenous immunoglobulins

### Comparison of clinical outcomes

In-hospital death was 30.8% (8/26) in patients not receiving IVIG and 22.7% (5/22) in patients treated with IVIG (Table 3). Median ICU length of stay was 11.5 days (IQR 3–19) for patients not treated with IVIG compared to 11 days (IQR 6–21) for patients treated with IVIG. Acute renal failure occurred in 50% (13/26) of patients not receiving IVIG and in 81.8% (18/22) of patients receiving IVIG. Patients not receiving IVIG developed ARDS in 23.1% (6/26) and patients receiving IVIG in 22.7% (5/22).

**Table 3:** Clinical outcomes according to group

	Non-IVIG (n = 26)	IVIG (n = 22)
Clinical outcomes		
In-hospital death	8/26 (30.8%)	5/22 (22.7%)
ICU length of stay (d)	11.5 (3–19)	11 (6–21)
Development of acute renal failure	13/26 (50%)	18/22 (81.8%)
Development of ARDS	6/26 (23.1%)	5/22 (22.7%)
Development of septic shock	12/26 (46.2%)	17/22 (77.3%)

Data expressed as numbers and percentage (%) or median and (interquartile range, IQR). ICU, intensive care unit; ARDS, acute respiratory distress syndrome; IVIG, intravenous immunoglobulins

Septic shock occurred in 12 of 26 (46.2%) patients not treated with IVIG and in 17 of 22 (77.3%) patients treated with IVIG (Table 3).

### Association between IVIG treatment and clinical outcomes

After adjustment for potential confounders including age, sex, affected body surface area and SAPS II score, no difference for in-hospital survival (hazard ratio 2.20, 95% confidence interval [CI] 0.24–20.20, p = 0.5), ICU length of stay (subhazard ratio [SHR] 0.90, CI 0.41–1.98, p = 0.8) and the development of ARDS (SHR 1.2, CI 0.36–4.03, p = 0.77) was observed in patients with or without IVIG treatment (Table 4). All patients received antibiotics, including beta lactams and clindamycin (Table 1). The risk of acute renal failure (SHR 2.86, CI 1.33–6.15, p = 0.01) and septic shock (SHR 1.86, CI 1.02–3.40, p = 0.04) was higher in patients treated with IVIG (Table 4).

**Table 4:** Treatment with intravenous immunoglobulins and clinical outcomes

Clinical outcomes	Adjusted HR/SHR^a^ (95% CI)
In-hospital death	2.20 (0.24 to 20.2)
ICU length of stay	0.90 (0.41 to 1.98)
Acute renal failure	2.86 (1.33 to 6.15)
ARDS	1.20 (0.36 to 4.03)
Septic shock	1.86 (1.02 to 3.40)

ICU, intensive care unit; ARDS, acute respiratory distress syndrome; HR, hazard ratio; SHR, subhazard ratio; CI, confidence interval

^a^For death a Cox regression model was used, resulting in a hazard ratio (HR) as estimate of effect, while for all other clinical outcomes the use of a competing risk regression model according to Fine and Gray resulted in subhazard ratios (SHR). All analyses were adjusted for age, sex, affected body surface area and simplified acute physiology score (SAPS) II at admission. Values < 1 correspond with a lower risk of the outcome in patients treated with intravenous immunoglobulins

## Discussion

In this retrospective study including 48 patients with necrotizing soft tissue infections (NSTI) due to various pathogens treated in a tertiary ICU from 2008 and 2020, no difference in mortality or length of ICU stay was observed in patients with or without IVIG treatment. Almost all patients received the recommended antibiotic combination of beta lactam plus protein synthesis inhibitors. However, patients receiving IVIG were at higher risk of acute renal failure and septic shock, possibly reflecting a higher disease severity beyond measured confounders.

Observational studies suggested an association between IVIG and prolonged survival after streptococcal infections [9–11, 20]. A prospective observational surveillance study involving 84 cases of invasive streptococcal infections suggested a positive effect of IVIG on survival in patients concomitantly receiving clindamycin [21]. Another prospective cohort study, involving more than 100 patients with streptococcal NSTI, found a higher mortality in patients who did not receive IVIG treatment [11]. Possible reasons for our findings are that our study did not only include streptococcal infections, but also patients with NSTI due to other microorganisms. Also, the patients in our cohort without IVIG treatment had a lower SAPS II score [11]. Further, overall mortality was relatively low in our study compared to other reports [1, 10], likely emphasizing the importance of high standard of care treatment modalities including early resuscitation, surgery and administration of antibiotics. In our cohort, the high-resource setting and the consequent early administration of high-dose clindamycin and broad-spectrum penicillins might have led to an overall low mortality, obfuscating the detection of a mortality benefit from IVIG.

In a randomised study including patients with NSTI and SAPS II scores similar to our patients, no effect of IVIG was found on the incidence of acute kidney injury, mechanical ventilation or decline of the Sequential Organ Failure Assessment (SOFA) score [22]. In our study, patients treated with IVIG were more likely to develop acute renal failure and septic shock, possibly reflecting a higher disease severity in the treatment group. IVIG was primarily administered to patients considered to be more severely ill, as indicated by the higher SAPS II score, C-reactive protein, creatinine and lactate dehydrogenase levels at ICU admission, indicating potential selection bias (Table 1). However, renal failure has also been described as a possible side effect of IVIG [23]. Possible pathophysiologic mechanisms include the precipitation of immune complexes in glomeruli, osmotic nephritis and immunological hemolysis-associated tubular obstruction [24]. Identified patient risk factors include pre-existing renal insufficiency, higher age, diabetes or hypovolemia [23]. Therefore, based on the above-mentioned risk factors and pathophysiological mechanisms, a causal relationship between the administration of IVIG and renal failure could explain the observed association in our patients. The increased baseline creatinine in patients receiving IVIG might have been a relevant risk factor. Further previously characterized adverse events of IVIG include the occurrence of altered consciousness, hypotension, tachycardia or anaphylactic reactions [24, 25]. In our cohort, the risk of septic shock was higher in patients receiving IVIG compared to patients not receiving IVIG. Based on certain clinical similarities between septic shock and the above-described adverse events (both can be categorized as forms of distributive circulatory compromise), we can’t exclude that the more frequent detection of shock in patients receiving IVIG in part might have been attributable to the administration of IVIG. Our finding thus highlights the importance of carefully balancing harms against potential benefits before administering IVIG in individual patients.

The missing association of IVIG treatment and length of ICU stay observed in our study has been described previously [9].

Our study has the strength of a long observation period of 13 years in a rather homogenous population from one tertiary care centre with high standard of care, surgical procedures and co-interventions. The competing risk of death was taken into account for non-death outcomes.

Our study also has several limitations. First, the retrospective observational design only allows for estimations of association, and not causality. Second, owing to the rare incidence of NSTI, the sample size was low, which limited the number of factors we could adjust for in our analyses and the precision of our estimates. Thus, residual confounding from unmeasured factors may bias our results, and power might have been too low to detect smaller treatment effects. Third, we included patients with NSTI due to various microorganisms, not focusing on streptococci alone, which might have influenced our findings. Fourth, inclusion of patients based on diagnosis codes and surgical reports might favour selection bias.

## Conclusions

In conclusion, we found no evidence for a clear benefit of IVIG treatment in NSTI in our cohort of NSTI due to various microorganisms. The fact that patients receiving IVIG appeared more severely ill warrants consideration of IVIG administration on a case-to-case basis (including considerations of possible harms, such as renal failure) in addition to the recommended antibiotic combination therapy including beta lactams and protein synthesis inhibitors. Adequately powered randomised controlled trials are needed to definitely determine the efficacy of IVIG in NSTI.

## Data Availability

The datasets used and/or analysed during the current study are available from the corresponding author on reasonable request.

## Abbreviations

ARDS: Acute respiratory distress syndrome
ICU: Intensive care unit
IVIG: Intravenous immunoglobulins
NSTI: Necrotizing soft-tissue infections
SAPS II score: Simplified Acute Physiology Score II
